# Supplementary material for: Transcriptomic Insights into Mechanisms of Early Seed Maturation in the Garden Pea (Pisum sativum L.)
Source: Cells. 2020 Mar 23;9(3):779. doi: 10.3390/cells9030779 (PMC7140803; doi:10.3390/cells9030779)

# Sprint−2(10) vs Zhewa

## Global Summary

%DE = 0.91

# genes with fdr < 0.2 = 575 ( 302 + / 273 −)

# genes with fdr < 0.1 = 538 ( 281 + / 257 −)

# genes with fdr < 0.05 = 520 ( 273 + / 247 −)

# genes with fdr < 0.01 = 369 ( 192 + / 177 −)

# genes in genesets = 0

<FC> = 0

<t−score> = −128.91

<p−value> = 0

<fdr> = 0.09

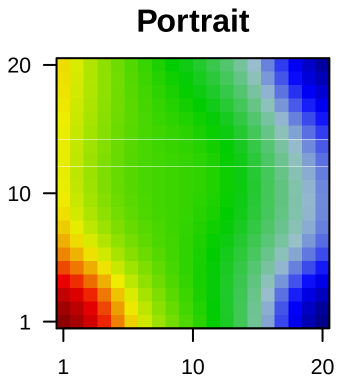


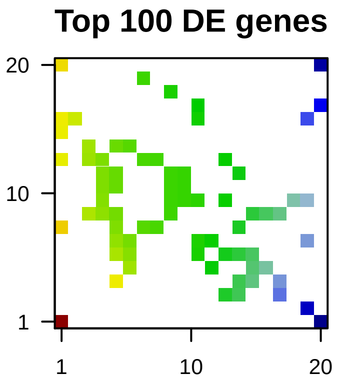


## Global Genelist

| Rank | ID | log(FC) | p−value | fdr | Metagene | Description |
|---|---|---|---|---|---|---|
| 1 | TRINITY_DN1 | −11.1 | 7e−15 | 6e−10 | 20 x 20 | |
| 2 | TRINITY_DN1 | −19.01 | 1e−11 | 8e−09 | 20 x 1 | |
| 3 | TRINITY_DN6 | −2.14 | 2e−10 | 2e−08 | 20 x 1 | |
| 4 | TRINITY_DN2 | −4.19 | 5e−10 | 5e−08 | 20 x 1 | |
| 5 | TRINITY_DN2 | −0.07 | 2e−09 | 5e−08 | 13 x 3 | |
| 6 | TRINITY_DN3 | −1.88 | 3e−09 | 5e−08 | 20 x 1 | |
| 7 | TRINITY_DN2 | −0.01 | 3e−09 | 1e−07 | 11 x 6 | |
| 8 | TRINITY_DN2 | −2.39 | 8e−09 | 1e−07 | 20 x 20 | |
| 9 | TRINITY_DN1 | −0.24 | 1e−08 | 1e−07 | 14 x 3 | |
| 10 | TRINITY_DN6 | −0.02 | 1e−08 | 1e−07 | 10 x 10 | |
| 11 | TRINITY_DN1 | −0.26 | 1e−08 | 1e−07 | 13 x 6 | |
| 12 | comp64083_c | −0.01 | 1e−08 | 1e−07 | 10 x 12 | |
| 13 | TRINITY_DN4 | 5.53 | 2e−08 | 3e−07 | 1 x 1 | |
| 14 | TRINITY_DN3 | 0.07 | 3e−08 | 3e−07 | 5 x 9 | |
| 15 | TRINITY_DN3 | −0.91 | 3e−08 | 1e−06 | 17 x 4 | |
| 16 | TRINITY_DN6 | −0.46 | 5e−08 | 1e−06 | 20 x 20 | |
| 17 | TRINITY_DN7 | −0.1 | 6e−08 | 2e−06 | 13 x 13 | |
| 18 | TRINITY_DN5 | −0.06 | 1e−07 | 2e−06 | 13 x 10 | |
| 19 | TRINITY_DN3 | −0.83 | 1e−07 | 2e−06 | 19 x 2 | |
| 20 | TRINITY_DN4 | 0.08 | 2e−07 | 2e−06 | 6 x 7 | |

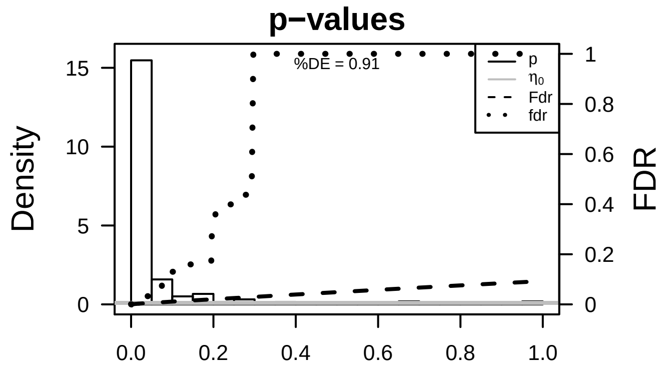

Supplement: Supplementary file 1 [file cells-09-00779-s001.zip › Supplementary materials/FileS3.zip!/FileS2/Hormones - Results/Summary Sheets - Differences/Reports/Sprint.2.10..vs.Zhewan.1.10..pdf]
